# Prevención de complicaciones y reducción de riesgos en implantología oral considerando los factores humanos y la conciencia de la situación

**DOI:** 10.21142/2523-2754-0901-2021-048

**Published:** 2021-03-11

**Authors:** Nadia Yudy González-Silva, Kevin Adonis Ronceros-Dueñas, Pedro Luis Tinedo-López, José Luis Huamaní-Echaccaya, María Eugenia Guerrero, Violeta Malpartida Carrillo

**Affiliations:** 1 Estudiante de pregrado, Escuela de Estomatología de la Universidad Privada San Juan Bautista. Lima, Perú. valeri18silva@hotmail.com, kevinadonisronce@gmail.com Universidad Privada San Juan Bautista Estudiante de pregrado, Escuela de Estomatología Universidad Privada San Juan Bautista Lima Peru valeri18silva@hotmail.com kevinadonisronce@gmail.com; 2 División de Periodoncia, Escuela de Estomatología de la Universidad Privada San Juan Bautista. Lima, Perú. pedro120488@outlook.es, viletayu_30@hotmail.com Universidad Privada San Juan Bautista División de Periodoncia, Escuela de Estomatología Universidad Privada San Juan Bautista Lima Peru pedro120488@outlook.es viletayu_30@hotmail.com; 3 División de Estomatología, Escuela de Estomatología de la Universidad Privada San Juan Bautista. Ica, Perú. odontoh1@gmail.com Universidad Privada San Juan Bautista División de Estomatología Escuela de Estomatología Universidad Privada San Juan Bautista Ica Peru odontoh1@gmail.com; 4 Departamento Académico Médico Quirúrgico, Facultad de Odontología de la Universidad Nacional Mayor de San Marcos. Lima, Perú. mega43@hotmail.com Universidad Nacional Mayor de San Marcos Departamento Académico Médico Quirúrgico Facultad de Odontología Universidad Nacional Mayor de San Marcos Lima Peru mega43@hotmail.com

**Keywords:** implantes dentales, complicaciones, riesgo a la salud, prevención de accidentes, dental implants, complications, health risk, accident prevention

## Abstract

En los últimos años, se ha incrementado el porcentaje de colocación de implantes dentales y, con ello, también la mayor presencia de eventos adversos, por lo que las complicaciones no son infrecuentes. La gran mayoría de recomendaciones para la reducción de complicaciones asociadas con la colocación de implantes dentales están analizadas desde el punto de vista de sus causas directas, juzgando la técnica o al individuo, pero no al sistema en conjunto, cuando este constituye la etiología real de las complicaciones. Recientemente, se ha empezado a considerar más importante la inclusión de los factores humanos y la conciencia de la situación en la comprensión de las complicaciones en implantología oral. Esto ha permitido analizar de manera global tanto al individuo como a su entorno, y aportar soluciones basadas en la prevención. Sin embargo, el conocimiento y la utilización de estos aspectos en implantología oral están aún en vías de popularización, por lo que el objetivo del presente artículo es difundir el enfoque de los factores humanos y la conciencia de la situación en la prevención de complicaciones y en la reducción de riesgos en los procedimientos de colocación de implantes dentales.

## Abstract (en)

In recent years, the percentage of dental implant placement has increased, and the presence of adverse events and complications are not uncommon. The vast majority of recommendations for reducing complications associated with dental implant placement are analyzed from the point of view of their direct causes, evaluating the technique and/or the individual, but not the system as a whole, being this the actual etiology of complications. The inclusion of human factors and situation awareness has recently gained increasing importance in oral implantology complications and has allowed global analysis of both the individual and the environment, providing solutions based on prevention. However, knowledge and use of these aspects in oral implantology are still in the process of popularization, and therefore, the objective of this article was to describe the human factors involved and situation awareness for preventing complications and reducing the risks associated with dental implant placement procedures.

## INTRODUCCIÓN

La rehabilitación oral con implantes dentales es considerada una opción de tratamiento predecible, con una tasa de éxito superior al 90% y una de supervivencia del 96,4% en estudios con hasta 10 años de seguimiento ^1^^,^^2^. Sin lugar a dudas, estos altos porcentajes dependen de una planificación prequirúrgica precisa, una técnica quirúrgica cuidadosa y un diseño protésico adecuado ^3^. Sin embargo, a pesar de los mejores esfuerzos del equipo de profesionales para proporcionar una atención óptima, ocasionalmente ocurren errores que afectan el éxito y la supervivencia de los implantes.

Los errores son impedimentos para los resultados exitosos de los tratamientos y tienen consecuencias importantes tanto para el paciente como para el profesional. En los pacientes, los errores conllevan a molestias considerables, angustias y pérdida de tiempo, ya que se requiere de visitas adicionales al dentista ^4^. Mientras tanto, los profesionales pueden enfrentar posibles demandas por inconformidades con el tratamiento y lesiones corporales, entre las más frecuentes pérdida de la sensibilidad, fístula oroantral y hemorragia potencialmente mortal ^5^. Por ende, la corrección de estos errores ocasiona pérdidas financieras significativas, así como consecuencias legales que pueden llegar incluso a la pérdida de la licencia ^6^.

En el 2017, Clark et al. ^7^ realizaron un estudio en tomografías computarizadas de haz cónico para identificar complicaciones poscolocación de implantes. Según los resultados de 2323 tomografías evaluadas, los autores reportaron un total de 160 (6,89%^)^ complicaciones relacionadas con el posicionamiento del implante. De ellas, 62 revelaron penetración del implante en alguna estructura anatómica adyacente. Específicamente, se reportaron 21 casos de penetración en el seno maxilar, 19 en la cavidad nasal, 9 en el conducto alveolar inferior y 13 en la cortical lingual. Asimismo, 15 casos de lesiones en dientes adyacentes. Con base en el considerable porcentaje de complicaciones, se recomienda que los cirujanos tengan una capacitación adecuada y una mejora continua de habilidades quirúrgicas para controlar y prevenirlas ^8^. Sin embargo, desafortunadamente, la mayoría de las recomendaciones para la reducción de los porcentajes de las complicaciones en implantología oral están analizadas desde el punto de vista de sus causas directas -se juzga la técnica o al individuo (potencialmente responsable de la complicación)-, pero no consideran al sistema en su conjunto, el cual constituye la etiología real de las complicaciones. 

Recientemente, se ha empezado a considerar con mayor realce la inclusión de los factores humanos en las complicaciones implantológicas dentales ^9^. Asimismo, la conciencia de la situación ^10^ se presenta como un enfoque que permite comprender las raíces reales del problema ya que analiza de manera global tanto al individuo como a su entorno, lo que aporta soluciones basadas en la prevención. Sin embargo, el conocimiento y la utilización de estos importantes aspectos en implantología oral aún están en vías de popularización.

De allí que el objetivo del presente artículo sea difundir el enfoque de los factores humanos y la conciencia de la situación para la prevención de complicaciones y la disminución de riesgos asociados con los procedimientos de colocación de implantes dentales.

### Fundamento

En los años 70 se produjo un gran avance en la seguridad de las personas en el campo de la aviación comercial, debido al análisis de accidentes aéreos catastróficos de la época. Los especialistas en aeronáutica dejaron de analizar el enfoque simplemente técnico y empezaron a buscar otras vías de mejora de la seguridad aérea. Los resultados revelaron que personas adecuadamente capacitadas y bien intencionadas eran capaces de cometer errores, es decir, se reconoció poco a poco que el error humano era la fuente de la mayoría de los accidentes ^11^. A partir de ello, la industria de la aviación ha adoptado el *crew resources management* o CRM (técnica de gestión que se basa en los factores humanos) como base para abordar los aspectos humanos en los vuelos ^10^. El CRM hace referencia al uso de toda la información de recursos, equipos y personas para lograr operaciones de vuelo seguras y eficientes, mediante la identificación temprana de errores ^12^. 

Según la literatura, pilotear un avión y practicar odontología son procedimientos altamente técnicos que requieren años de entrenamiento y de práctica para alcanzar altos niveles de competencia. Se considera que la cabina de vuelo y el consultorio dental tienen muchas similitudes, ya que la experiencia, la información, el equipo y las personas son esenciales para el éxito del vuelo, así como para los resultados clínicos odontológicos ^13^. El enfoque CRM se ha extendido a otras áreas como la industria nuclear, el transporte ferroviario y pluvial, y ha logrado que las actividades humanas en estos ámbitos sean cada vez más seguras ^9^^,^^10^. Además, se ha implementado su uso en el área de la salud. Sin embargo, la profesión odontológica aún no ha incorporado elementos esenciales del CRM en sus especialidades ^13^, por lo que es necesario incrementar su conocimiento y difusión.

### Seguridad y riesgos. Fallos, errores y complicaciones

El diccionario define *seguridad* como “la condición de estar a salvo de sufrir o causar lesiones o pérdidas”, mientras que *riesgo* es la “posibilidad de alguna pérdida o lesión” ^14^. Se brinda seguridad en los tratamientos mediante el cumplimiento de protocolos efectivos y la identificación temprana de posibles riesgos y errores. Se considera fallo a la desviación voluntaria de las reglas de seguridad conocidas, es decir, cuando la persona actúa ignorando la seguridad por conveniencia personal y esto puede estar ligado a una actividad inadecuada, como la negligencia. Por otro lado, el error es el resultado de una acción distinta de lo esperado, lo que produce una divergencia entre el objetivo del procedimiento y el resultado obtenido. Se considera que el error es indisociable de la condición humana ^10^.

Debido a que el error humano es inevitable, es imposible practicar la odontología y sus especialidades sin cometer errores, aunque lo más frecuente es que estos no provoquen complicaciones. Una estimación no procedente de la literatura describe que un 90% de los errores son absorbidos por el sistema ^10^, es decir, son solucionados en el transcurso del procedimiento. Si bien los odontólogos han desarrollado sus propias estrategias para mitigar los errores, se ha notado poco esfuerzo por formular soluciones sistemáticas considerando la conciencia de la situación.

**Figura 1:**
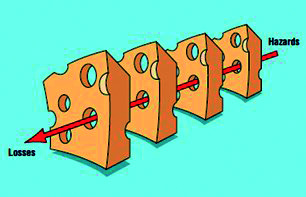
Modelo del queso suizo propuesto por Reason.

**Figura 2:**
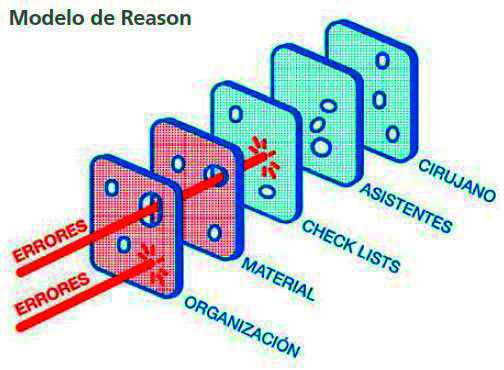
El establecimiento de barreras de protección evitará consecuencias graves de los errores. Fuente: Renouard et al.

**Figura 3:**
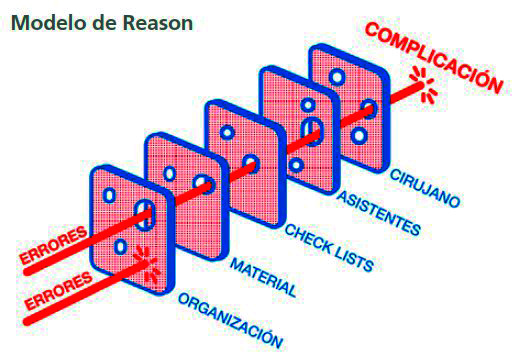
Alineamiento de circunstancias que propician la trayectoria directa de los errores hacia alguna complicación debido a la ausencia de barreras de protección. Fuente: Renouard et al.

La transformación de un error en una complicación se explica según el *swiss cheese model* (modelo del queso suizo) propuesto por Reason en el año 2000 para explicar los accidentes (Fig. 1) ^15^. Este modelo se basa en establecer barreras de protección sucesivas a modo de rebanadas de queso que tienen muchos agujeros, aunque a diferencia del queso estos agujeros se abren, cierran y cambian de ubicación continuamente (Fig. 2). La presencia de agujeros en cualquier barrera de protección normalmente no causa un mal resultado, a menos que se dé un alineamiento de agujeros que permita la trayectoria directa de los errores hacia una complicación (Fig. 3).

### Los factores humanos y la conciencia de la situación

Las publicaciones recientes sobre la inclusión del enfoque CRM en implantología oral han sido liderados por el equipo del Dr. Franck Renouard ^9^^,^^10^. Según sus investigaciones, los fracasos en el campo de la implantología oral son generalmente analizados desde el punto de vista técnico considerando complicaciones mecánicas y biológicas ^9^; sin embargo, el rol que cumplen el cirujano y su equipo de trabajo en el éxito o el fracaso de estos procedimientos ha sido poco considerado ^16^^,^^17^. A veces, se analizan desde un punto de vista reprensivo, que busca causas directas y analiza responsabilidades que se enfocan en los autores más próximos (cirujanos^)^ a los acontecimientos indeseables o fracasos, pero casi nunca comprenden las raíces reales del problema, ya que no analizan de manera global tanto al individuo como a su entorno ^9^^,^^10^. La consideración de los factores humanos en las complicaciones ha permitido entender que las causas de los acontecimientos indeseables o eventos adversos no están frecuentemente relacionadas con la fata de experiencia de un profesional capacitado, sino con la incapacidad de aplicar el conocimiento adecuadamente debido a un ambiente adverso o a un nivel de estrés elevado ^9^.

Según se indica, la competencia constituye todo el conocimiento y la experiencia que posee un individuo en un momento dado de su carrera. Por otro lado, el desempeño es la habilidad con la que este individuo emplea su conocimiento y experiencia en un ambiente y un momento específicos. Se puede entender que el desempeño individual de un cirujano puede cambiar si está cansado, fatigado, estresado o distraído por temas personales, pese a que su nivel de competencia sea el mismo. Por lo tanto, las complicaciones no están relacionadas con la falta de conocimiento, sino con una mala o nula utilización de esos conocimientos ^18^^,^^19^.

Un punto importante por considerar es el estrés. Para los humanos, el 90% del estrés es endógeno o autoinducido, es decir, un producto de la imaginación en lugar de las circunstancias actuales. Ciertamente, la percepción de la situación es la que conlleva a modificaciones del comportamiento y resulta importante reconocer las señales de advertencia, entre ellas el aumento de la frecuencia cardíaca y respiratoria, los diversos grados de temblores, los cambios en el color de la piel (palidez, enrojecimiento) y la sequedad de la boca ^9^. Renouard et al. ^10^ describieron un fenómeno ligado al estrés durante el procedimiento quirúrgico denominado “tunelización mental”. Según estos autores, la esta se produce cuando el cirujano se queda anclado en su objetivo final, pase lo que pase, con el fin de huir lo más rápido posible de la situación en que se encuentra y continúa haste llegar a una complicación como consecuencia de la incapacidad de adaptarse a la situación a por el estrés acumulado. Según se cita en un artículo de Renouard et al. ^9^: “Como la corteza prefrontal no puede manejar varias ideas al mismo tiempo, no es posible dedicar toda su atención a la cirugía si, por ejemplo, al mismo tiempo, uno se preocupa de que el próximo paciente llegue tarde”. Esta sobrecarga cognitiva es una fuente importante de estrés.

En el campo de la aviación se identifican cinco actitudes o comportamientos peligrosos que incrementan el riesgo de complicaciones: la impulsividad, el rechazo a la autoridad, la invulnerabilidad, el machismo y la resignación (Tabla 1) ^20^). Las primeras cuatro actitudes llevan al individuo a “bajar la guardia”, mientras que la resignación resulta en la extrema precaución por el temor de poner en riesgo al paciente con posibles fallas. Claramente, estas actitudes o comportamientos pueden ocurrir al llevar a cabo algún procedimiento quirúrgico de nuestra especialidad.

**Tabla 1:** Cinco actitudes/comportamientos peligrosos. Fuente: Renouard y Charrier ^20^

ACTITUD	CARACTERÍSTICA
Impulsividad	“Rápido, rápido, rápido”. Los profesionales impulsivos sienten la necesidad de hacer todo rápidamente. Solo piensan en lo que van a hacer e inmediatamente hacen lo primero que se les ocurre.
Rechazo a la autoridad	“No me digas lo que tengo que hacer”. Los profesionales afectados por una actitud de rechazo a la autoridad piensan que las reglas, las regulaciones y los procedimientos son inútiles o no están diseñados para ellos. Piensan que nadie tiene derecho a decirles cómo comportarse. Esta actitud es bastante común entre los profesionales que generalmente trabajan solos, como los dentistas.
Invulnerabilidad	“Eso no podría pasarme a mí”. Algunas personas piensan que los accidentes solo les ocurren a otros. Este sesgo analítico afecta a todos hasta cierto punto, pero puede ser particularmente marcado en ciertos individuos.
Machismo	“Yo puedo hacer esto”. Los profesionales con esta actitud intentan demostrar su superioridad sobre los demás. Aunque esta es una actitud predominantemente masculina, también puede afectar a las cirujanas.
Resignación	“De qué sirve…”. Los profesionales afectados por la resignación no creen que sus acciones hagan alguna diferencia en cuanto a si un resultado es un éxito o un fracaso. A veces, este tipo de profesionales cede ante demandas irracionales de los pacientes solo para ser “amables”.

La conciencia de la situación se define como la capacidad de entender el entorno y anticiparse a sus variaciones. Es una herramienta que proviene de la aviación comercial y es útil para abordar la etiología de las complicaciones, al conocer sus causas profundas y originales. Por ello, la OMS la califica como crucial en todos los ámbitos sanitarios ^10^. Se divide en tres niveles: la percepción de la información, relacionada con diferenciar lo que es crucial de lo que no; la comprensión de la información, es decir, darle sentido a la información analizando los errores latentes; y la anticipación y adaptación a la situación, relacionada con el conocimiento y establecimiento de un plan alterno o plan B ^10^. También bajo este enfoque se recomienda reforzar las medidas preventivas de seguridad utilizando listas de verificación (*checklist*) preoperatorias de manera sistemática, ya que esto permitirá la detección de infracciones o contraindicaciones antes de empezar la cirugía.

La conciencia de la situación permite analizar retrospectivamente las complicaciones de una manera muy eficaz. El objetivo no es librar a los clínicos de sus responsabilidades, sino intentar comprender las raíces reales del problema para aportar soluciones en su prevención ^10^.

### Prevención de complicaciones y reducción de riesgos en implantología oral

Durante los últimos años se ha observado un gran incremento en el porcentaje de colocación de implantes dentales y, con ello, también la presencia de mayores eventos adversos, por lo que las complicaciones relacionadas con los implantes dentales no son infrecuentes ^7^. En la literatura se mencionan diversas clasificaciones de complicaciones en implantología oral, entre las que se encuentran las complicaciones operatorias e inflamatorias ^21^; intraoperatorias, postoperatorias, y protésicas-mecánicas ^22^; de tejidos blandos y de tejidos duros ^23^; relacionadas con el plan de tratamiento, la anatomía, el procedimiento y otros factores ^24^; tempranas y tardías ^25^; quirúrgicas comunes y no comunes ^26^; y relacionadas con factores internos y externos ^27^. Todas estas clasificaciones incluyen recomendaciones para la prevención y el tratamiento, mientras que solo dos hacen referencia específica a las posibles causas ^(22, 24)^. Asimismo, apenas una considera a la yatrogenia y al error humano como posible causa de complicaciones, incluso presenta una lista de verificación para prevenirlas y manejarlas ^24^. 

Por otro lado, uno de los Informes Científicos del Congreso de la 27 Reunión Científica del EAO (European Association for Osseointegration), celebrada en Viena el 2018, menciona que el enfoque actual en implantología oral es la prevención y la reducción de riesgos ^28^. Este enfoque actual se centra específicamente en el trabajo digital, la cirugía sin colgajo y el uso de materiales para la regeneración ósea guiada. 

En cuanto al trabajo digital, se recomienda disipar la idea de que lo digital es infalible, ya que existen posibilidades de error y riesgos en su aplicación. En la fase de adquisición de los datos, existen tres fuentes principales de posibles errores: movimiento del paciente, artefactos metálicos y posición incorrecta de la plantilla radiológica. Asimismo, muestran más riesgo los casos que involucran pacientes totalmente edéntulos y los que presentan apertura bucal limitada. 

La cirugía sin colgajo se ha presentado como una opción quirúrgica mínimamente invasiva, debido a que es un procedimiento menos extenso, pero se trata de una técnica ciega, lo que aumenta el riesgo de perforar las placas corticales y colocar los implantes fuera del reborde alveolar. Incluso, en el caso de procedimientos sin colgajo guiados mediante trabajo digital, el riesgo de perforación persiste debido a desviaciones inevitables que ocurren durante la cirugía guiada. Se recomiendan enfoques sin colgajo solo en casos en que las condiciones sean adecuadas por el grosor del hueso y una cantidad suficiente de encía.

Para lograr resultados óptimos, el 40% de los casos requieren procedimientos de aumento de tejido duro o blando antes de colocar los implantes, y este porcentaje se incrementa al 90% en las zonas estéticas. Los riesgos asociados con el aumento óseo dependen de una serie de factores, la mayoría de los cuales son más relevantes que las propiedades de los materiales de regeneración ósea utilizados. Estos factores incluyen el estado inmunológico, las enfermedades sistémicas, la higiene bucal, el estilo de vida y el cumplimiento del paciente. Además, la morfología del defecto, la cantidad y calidad del hueso y el tipo de tratamiento (colocación inmediata, carga, etc.) que se utiliza. La descelularización no puede garantizarse en el 100% de los casos, ya que pueden encontrarse pequeños restos de ADN, pero que no han demostrado ninguna antigenicidad. Se recomienda que este riesgo teórico se incluya en la información preoperatoria del paciente, aunque no existen casos documentados en el campo odontológico. 

Según el informe del congreso, los riesgos asociados con el procedimiento de colocación de implantes solo pueden manejarse de manera segura después de que se elabore un plan de tratamiento adecuado e individualizado, es decir, entender que “no planificar es planear para fallar”

## CONCLUSIONES

Los factores humanos y la conciencia de la situación se presentan como enfoques importantes para prevenir complicaciones y reducir riesgos en implantología oral. Considerar los factores humanos permite entender que, en el caso de un profesional capacitado, los eventos adversos no están frecuentemente relacionados con la fata de experiencia, sino con la incapacidad de aplicar el conocimiento adecuadamente debido a un ambiente adverso o un nivel de estrés elevado. La conciencia de la situación permite establecer una cultura real de seguridad mediante la aplicación de barreras de protección representadas por el uso de de listas de verificación y el conocimiento de un plan alterno para la solución de complicaciones. Estos enfoques deben ser considerados en la enseñanza de futuros especialistas, así como en las prácticas clínicas privadas y servicios hospitalarios, con la finalidad de prevenir complicaciones y reducir riegos.

## Fuente de financiamiento:

Autofinanciado
